# Method for the location of primary wear scars from retrieved metal on metal hip replacements

**DOI:** 10.1186/s12891-015-0622-2

**Published:** 2015-07-30

**Authors:** Garima Govind, Johann Henckel, Harry Hothi, Shiraz Sabah, John Skinner, Alister Hart

**Affiliations:** University College London, Institute of Orthopaedics and Musculoskeletal Science, Royal National Orthopaedic Hospital, Brockley Hill, Stanmore, HA7 4LP UK

**Keywords:** Joint mechanics, Hip arthroplasty, Wear scar, Acetabular cup

## Abstract

**Background:**

Retrieved metal-on-metal acetabular cups are valuable resources in investigating the wear behaviour of failed hip implants, but adequate methods to do so are lacking. To further contribute to addressing this issue, we developed a method to detect the *in vivo* location of the primary wear scar of an explanted cup.

**Methods:**

We proposed a new method in which thirteen patients with failed metal hip resurfacings were recruited, and their acetabular components retrieved. A 3D wear map was generated and the precise location of the primary wear scar in each cup was identified using a coordinate measuring machine. This wear scar location was noted in relation to the features on the acetabular cup. Having identified the location of the wear scar, this 3D positional map was co-registered to the implant on the patient’s pelvic 3D CT scan.

**Results:**

Using our proposed technique, we were able to demonstrate that the *in vivo* position of the primary wear scar in explanted metal acetabular cups can be variable.

**Conclusions:**

This method has utilised existing techniques to better understand the three-dimensional properties of wear behaviour, and may be a method which can be used in further studies to investigate variables that affect the position of the primary wear scar.

## Background

Metal-on-metal (MoM) hip implants proved to be popular, but had a high failure rate, and lead to a dangerous level of metal ions into the blood stream [1–4]. With patient factors a very important consideration, the pattern of failure due to biomechanical implications as a result of the patient’s anatomy must be examined [5, 6]. Pin pointing the *in vivo* location of the primary wear scar (WS_1_) may allow us to correlate a number of factors to help ascertain the tribology behind qualitative patterns of wear.

Limited work has been previously done to identify the location of the WS_1_ in explanted acetabular components of a MoM hip replacement with its *in* vivo location. Published work exploring the significance of wear scar location has so far been limited to an *in vitro* study [7]. In this study, Angadji et al. used schematic drawings to map two-dimensional acetabular wear scar locations. However as this study was done using a hip joint simulator, there was no method that described the process of co registering the WS_1_ with its *in vivo* location.

This limited previous work highlights the methodology is missing in this discipline, and our study aimed to address this gap in both technique and knowledge of *in* vivo wear scar location. Ours was a study aimed to develop a novel technique which allowed the mapping of a primary wear scar with its 3D pre-explantation *in vivo* location.

## Methods

As the purpose of this paper was to devise a novel technique that could use explanted acetabular cups to map the WS_1_ with its pre-explanation *in vivo* location, the method consisted of two main stages: retrieval analysis and 3D CT co-registration.

### Patients

CT scans for thirteen patients (Table 1) who had undergone a revision of their hip resurfacing that had subsequently failed was gathered. The two inclusion criteria were that the acetabular component needed to be available for physical analysis and that pre-revision CT scans of the patients were required. The mean age of the patients at the time of failure was 51.8 years and the mean period of between implant insertion and failure was 47.5 months. Twelve patients had been fitted with a Birmingham Hip Replacement (BHR) system. One patient had a Cormet system. Four patients had an implant on their left hip. The remaining nine patients had an implant on their right hip. One patient had a bilateral BHR but only the right hip was used for the purposes of this study. The internal diameter ranged from 42-54mm (Table 1). Informed written consent for this study was obtained from all patients.

**Table 1:** A summary of the patient demographics and implant details used in this study

Patient	Implant type	Femoral head radius (mm)	Acetabular cup radius (mm)	Age at insertion (years)	Time implanted (months)
1	BHR	50	56	45	23
2	BHR	46	54	50	25
3	BHR	50	56	68	60
4	BHR	46	52	54	75
5	BHR	42	50	57	56
6	BHR	42	48	59	31
7	BHR	42	48	46	63
8	BHR	50	56	56	84
9	BHR	42	50	55	44
10	BHR	42	50	38	33
11	BHR	54	60	63	63
12	BHR	54	60	47	23
13	Cormet	52	58	36	37

### Retrieval analysis

**Fig. 1:**
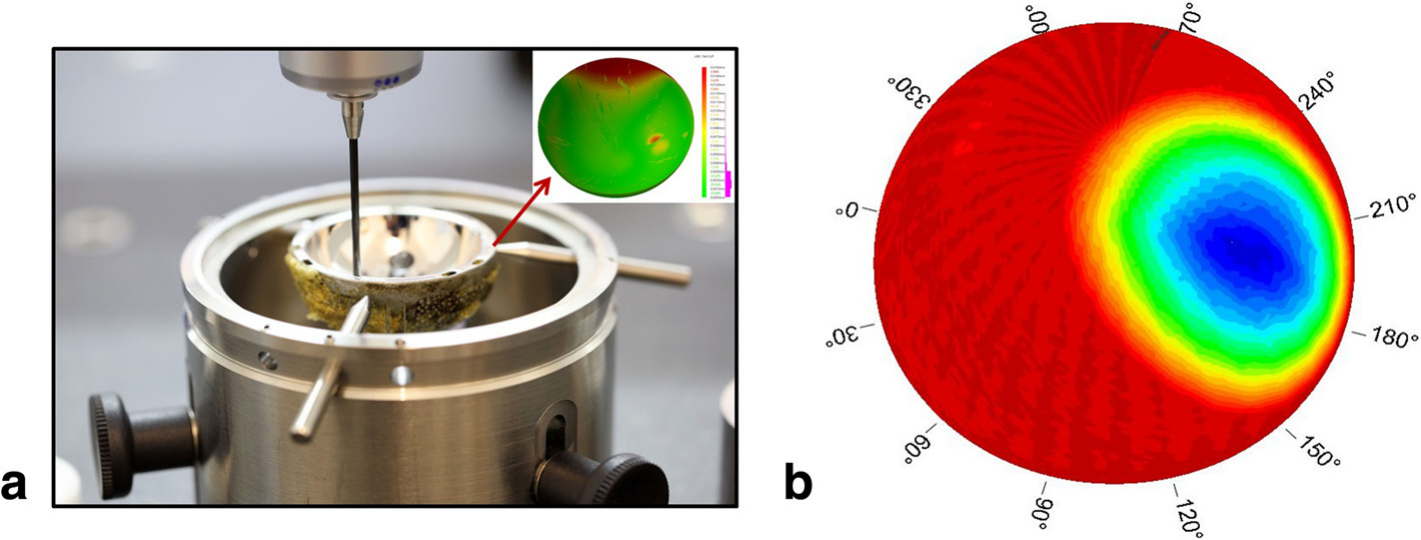
An example of a CMM and a wear map. **a** An acetabular cup placed in a coordinate measuring machine, with its corresponding 3D wear map in the inset (**b**) a close up of the 3D wear map, showing the wear scar location

**Fig. 2:**
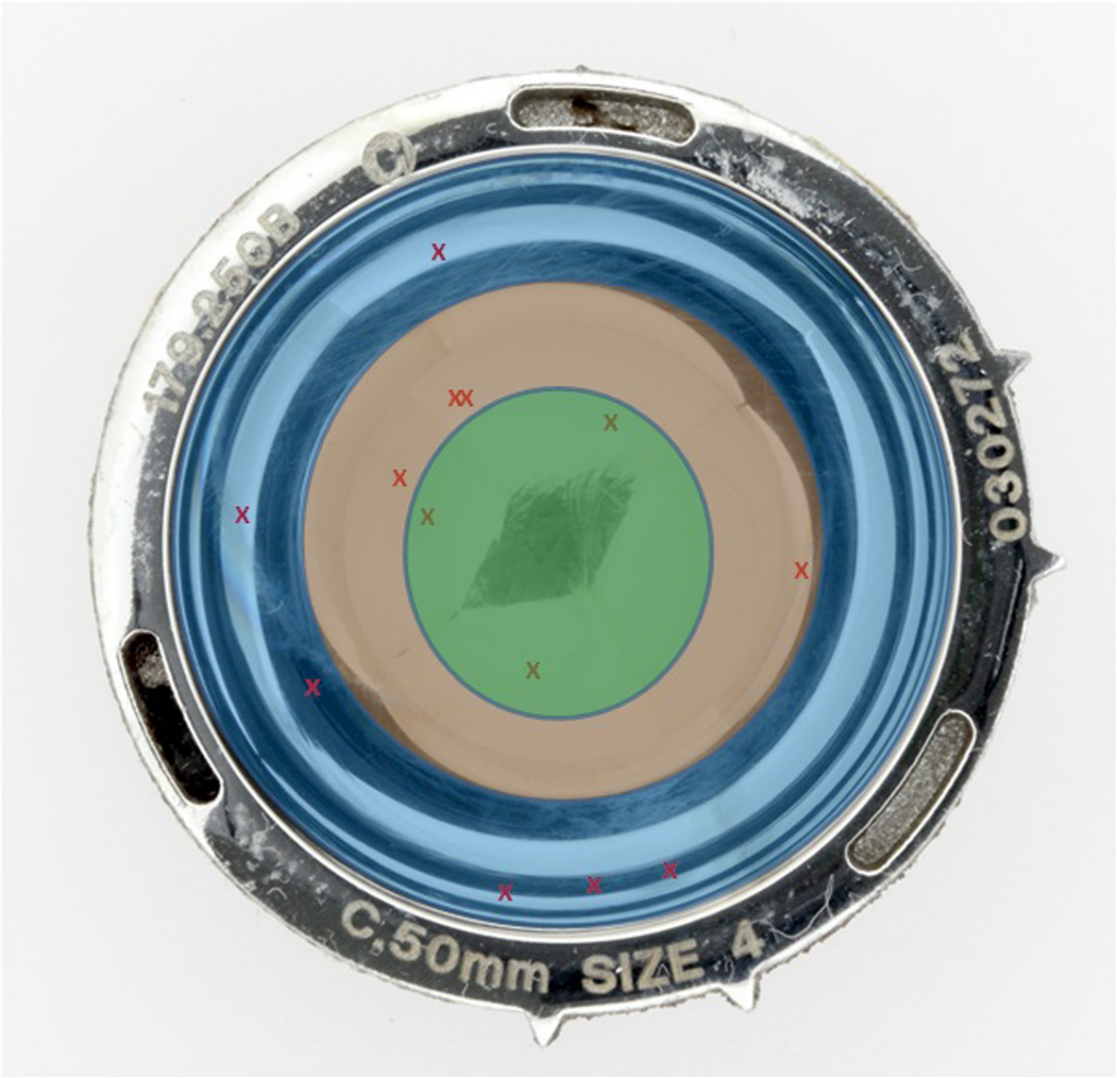
Distribution of primary wear scars. The schematic distribution of the 13 wear scars superimposed on a picture of a metal acetabular cup and arranged according to whether they are found in the outer (blue), middle (orange) or inner (green) zones

**Fig. 3:**
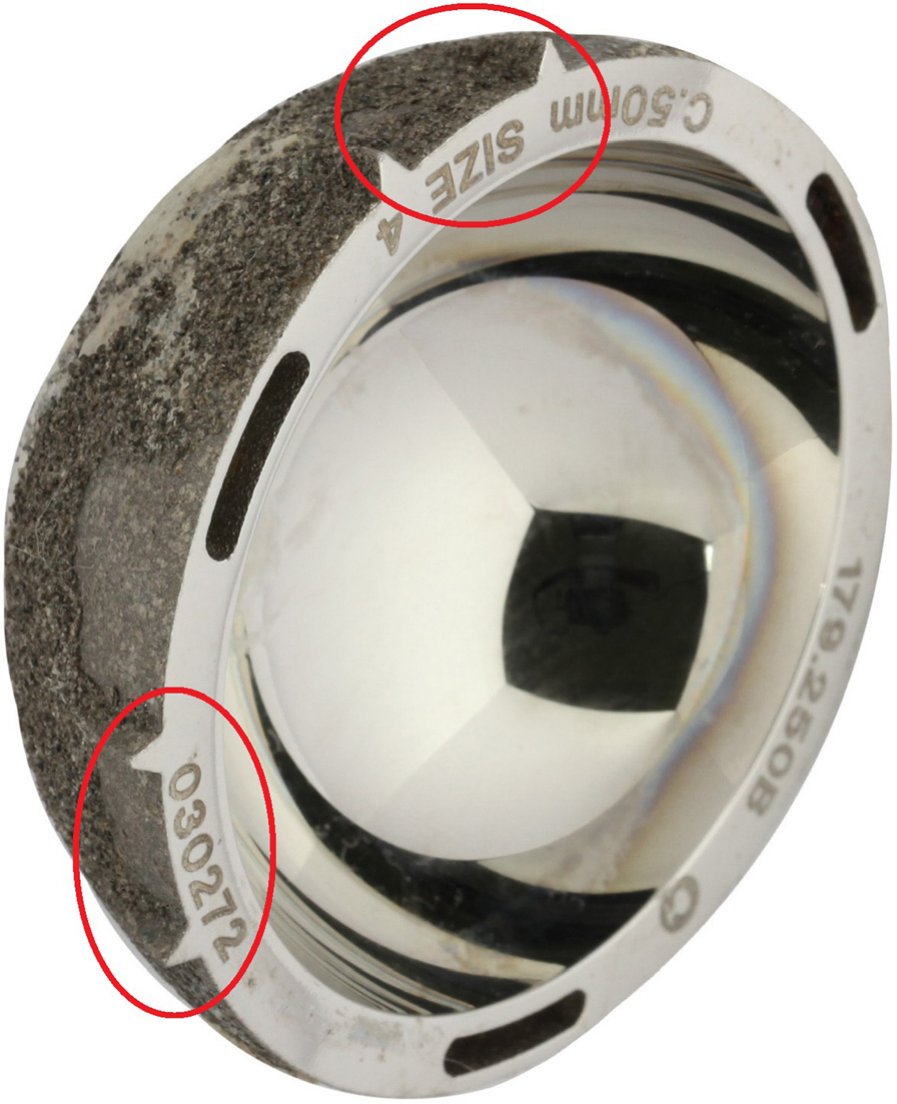
Acetabular features. An image of a metal acetabular cup, clearly showing the acetabular features which are circled in red

To determine the location of the wear scar, each of the bearing surfaces of the cup were measured using a Zeiss Prismo (Carl Zeiss Ltd, Rugby, UK) coordinate measuring machine (CMM). Previously published protocols [8] were used to take up to 300 000 unique data points on the surface by translating a 2mm ruby stylus along 400 polar scan lines. An iterative least square fitting method was used to analyse the raw data, allowing a 3D wear map of the cup under study to be generated (Fig. 1). An unworn cup should have had a uniform depth throughout the cup, and any areas of increased depth reflected locations of wear patches. The WS_1_ could be visualised according to their location according to concentric zone (Fig. 2). The location of WS_1_ was also noted in terms of degrees clockwise from the acetabular features (Fig. 3) which were used as markers for rotational identification.

### 3D CT and co-registration

**Fig. 4:**
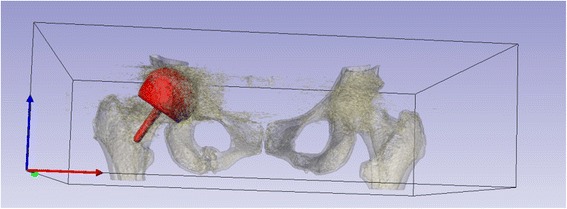
3D CT methodology. An example of the co-registration process within the software used, showing the *in vivo* position of the implant within the pelvis

A low dose 3DCT protocol was followed [9], generating an image of the pelvis. The *in vivo* location of WS_1_ could be co-registered on this image as the location of the WS_1_ was known in relation to the acetabular cup rim and also in relation to the acetabular features. First, the acetabular features were located on the 3D CT image. Next, the angle in degrees between these fins and the WS_1_ was measured using the appropriate measuring tools. Finally, the vertical distance of the WS_1_ from the acetabular cup rim was measured. This gave us the epicentre of the WS_1_ and hence, the location of the WS_1_ of an explanted acetabular cup could be co-registered with its *in vivo* location (Fig. 4).

Acetabular fins are intended add to the component’s stability [10], but in this case they could be used as a reference point in this co-registration process. Due to the spherical nature of the acetabular cup, without these acetabular fins the orientation of the explanted cup *in vivo* would not have been possible.

### Ethical approval

Ethical approval for this project was granted for this project in 2009 by the Integrated Research Application System Research Ethics Committee (number 07/QQ0401/25).

## Results

**Fig 5:**
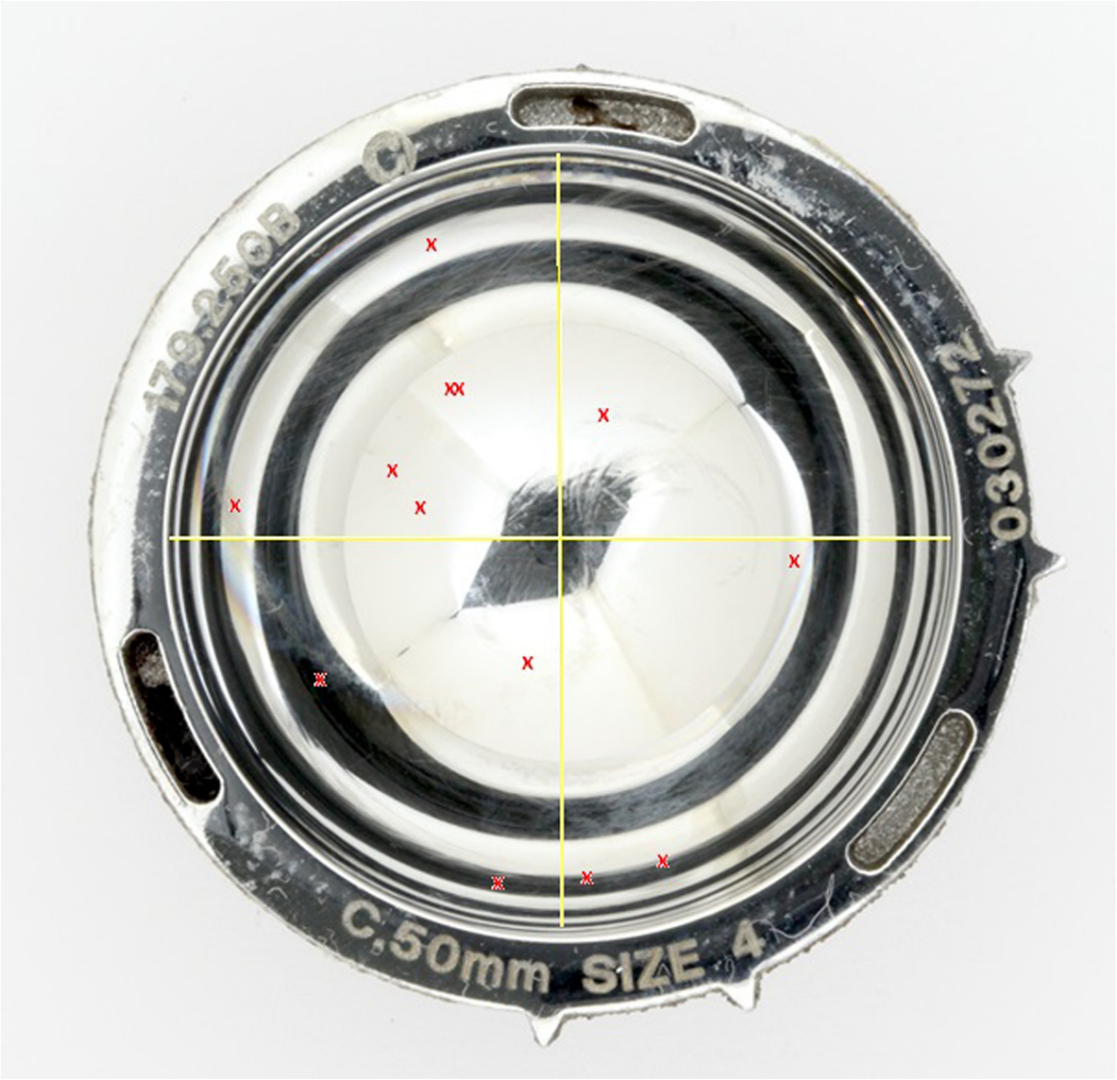
Location of primary wear scars. An image of a metal cup on which each of the 13 wear scars is superimposed, according to which quadrant the wear scar was positioned

Using the method which was discussed the WS_1_ was identified for all 13 components. The out-of-roundness machine was able to take circular measurements at 0.1° intervals around the acetabular cup rim and also in 0.5mm increments down from the acetabular cup rim. The location of the acetabular features was also noted successfully in all cases, allowing the co-registration of the *in vivo* location of the WS_1_ to be visualised. A diagram was then produced showing a 3D representation the location of each of the thirteen WS_1_ on a single acetabular cup. This allowed us to appreciate the range of their locations, with reference to the anterior pelvic plane, according to quadrants (Fig. 5).

## Discussion

The distribution of the location of the *in vivo* WS_1_ can be reported as follows: six were in the upper outer quadrant; three were in the lower outer quadrant; one was in the upper inner quadrant; and three were in the lower inner quadrant. These results clearly show that the location of the *in vivo* WS_1_ can be very variable, which does not fit with the current theory of edge loading [6, 7]. Further work can be done to correlate a range of factors with the location of WS_1_, and as such, the method devised here may be of huge importance. We did not encounter any components where this technique could not be used.

This is a novel method because it allows us to use an explanted component and work retrospectively to co-register the WS_1_ from this explanted component with what would have been its *in vivo* location. Mapping of wear scars of large numbers of failed components, and hence the application of our technique, may help us better investigate the qualitative three-dimensional properties of wear behaviour and correlate the location of wear with a number of variables. Knowledge of *in vivo* wear scar location may be key to assessing the importance of patient factors on wear properties and failure patterns.

The clinical value of the information obtained from this method will be of interest to surgeons who can suggest a more accurate patient-based prediction of implant longevity pre-revision surgery. Furthermore, it will be of use in the context of explaining wear behaviour of hip implants because the effect and importance of many variables are currently unknown. This is because of the limited investigations gathered out with respect to the qualitative properties of hip wear, such as primary wear scar location.

The mean period of implant failure is just under four years for our patient sample. This short length of time between implantation and failure may be explained by a selection bias, as we did not include patients whose implants had not failed, even though the implant may have had a substantial wear scar. Hence, an interesting aspect that has been unexplored in this study is whether there is a correlation between the period of implant insertion and the magnitude of the wear scar, This was one limitation of the study and in future, a method may be devised that allows us to visualise the *in vivo* primary wear scar pre-failure.

## Conclusions

We have developed a method which can be used as part of future mechanical and tribological studies looking into the failure of MoM hip implants. Future studies should investigate variables that affect position of the WS_1_, and our technique may be integral to these studies. Examples of variables could include component position, pelvic tilt, gender and joint reaction force. In doing so, we may be able to better understand the implant, surgical and patient factors that lead to implant failure and use this knowledge to predict the suitability of patients to certain joint replacement procedures and its prognosis.

## Abbreviations

MoM: Metal-on-metal
WS_1_: Primary wear scar
BHR: Birmingham Hip Replacement
CMM: Coordinate measuring machine
